# Purely antiferromagnetic magnetoelectric random access memory

**DOI:** 10.1038/ncomms13985

**Published:** 2017-01-03

**Authors:** Tobias Kosub, Martin Kopte, Ruben Hühne, Patrick Appel, Brendan Shields, Patrick Maletinsky, René Hübner, Maciej Oskar Liedke, Jürgen Fassbender, Oliver G. Schmidt, Denys Makarov

**Affiliations:** 1 Institute for Integrative Nanosciences, Institute for Solid State and Materials Research (IFW Dresden e.V.), 01069 Dresden, Germany; 2 Helmholtz-Zentrum Dresden-Rossendorf e.V., Institute of Ion Beam Physics and Materials Research, 01328 Dresden, Germany; 3 Institute for Metallic Materials, Institute for Solid State and Materials Research (IFW Dresden e.V.), 01069 Dresden; 4 Department of Physics, University of Basel, 4056 Basel, Switzerland; 5 Helmholtz-Zentrum Dresden-Rossendorf e.V., Institute of Radiation Physics, 01328 Dresden, Germany

## Abstract

Magnetic random access memory schemes employing magnetoelectric coupling to write binary information promise outstanding energy efficiency. We propose and demonstrate a purely antiferromagnetic magnetoelectric random access memory (AF-MERAM) that offers a remarkable 50-fold reduction of the writing threshold compared with ferromagnet-based counterparts, is robust against magnetic disturbances and exhibits no ferromagnetic hysteresis losses. Using the magnetoelectric antiferromagnet Cr_2_O_3_, we demonstrate reliable isothermal switching via gate voltage pulses and all-electric readout at room temperature. As no ferromagnetic component is present in the system, the writing magnetic field does not need to be pulsed for readout, allowing permanent magnets to be used. Based on our prototypes, we construct a comprehensive model of the magnetoelectric selection mechanisms in thin films of magnetoelectric antiferromagnets, revealing misfit induced ferrimagnetism as an important factor. Beyond memory applications, the AF-MERAM concept introduces a general all-electric interface for antiferromagnets and should find wide applicability in antiferromagnetic spintronics.

## Abstract (web summary)

Magnetoelectric coupling allows switching of magnetic states via gate voltage pulses. Here the authors propose and demonstrate a purely antiferromagnetic magnetoelectric random access memory based on Cr_2_O_3_, reporting 50-fold reduction of writing threshold compared to ferromagnetic counterparts.

In the effort to develop low-power data processing and storage devices, nonvolatile random access memory schemes have received considerable attention^1^. Magnetic elements such as the magnetic random access memory (MRAM) (Fig. 1a) promise excellent speed, superior rewritability and small footprints, which has led to strong commercial interest in this technology for memory applications. In addition to ferromagnetic MRAM, two complementary approaches have recently emerged for advancing beyond conventional MRAM elements in terms of its writing power and data robustness. On the one hand, switching and reading the antiferromagnetic order parameter of metallic antiferromagnets with charge currents^2^,^3^ has enabled purely antiferromagnetic MRAM (AF-MRAM), granting superior data stability against large magnetic disturbances and potentially even faster switchability. On the other hand, magnetoelectric random access memory (MERAM) promises energy efficient writing of antiferromagnets, by eliminating the need for charge currents through the memory cell and instead relying on electric field-induced writing. Reading out the antiferromagnetic state from MERAM has presented a challenge to date as magnetoelectric antiferromagnets (for example, BiFeO_3_ or Cr_2_O_3_) are dielectrics. Therefore, the readout signal of MERAM cells is conventionally acquired from a ferromagnet that is coupled with the magnetoelectric antiferromagnet by exchange bias^4^,^5^,^6^,^7^,^8^. While ferromagnets enable readability, their presence strongly interferes with the magnetoelectric selection of the antiferromagnetic order parameter^9^. This is related to exchange bias and ferromagnetic hysteresis, both of which need to be overcome in the writing process of MERAM with ferromagnets.

Here we put forth the concept of purely antiferromagnetic MERAM (AF-MERAM) (Fig. 1a), which avoids the issues associated with the presence of ferromagnets by instead using polarizable paramagnets, for example, Pt, to probe the order parameter of the magnetoelectric antiferromagnet. As shown schematically in Fig. 1b, the prototypical memory cell consists of an active layer of insulating magnetoelectric antiferromagnet, a bottom gate electrode for writing purposes and a top electrode that provides the readout interface via anomalous Hall measurements^10^. Using Cr_2_O_3_ as an AF element, we demonstrate a complete working AF-MERAM cell, proving that this concept yields substantial improvements in terms of magnetoelectric performance over comparable MERAM realizations with ferromagnets. In particular, by removing the ferromagnetic component from MERAM, we reduce the writing threshold by a factor of about 50. These characteristics render AF-MERAM a promising new member to the emerging field of purely antiferromagnetic spintronics^3^,^11^. We show the magnetoelectric writing and all-electric reading operations of a cell at room temperature over hundreds of read–write cycles. While nonvolatile solid-state memory is one possible application of AF-MERAM cells, the concept is applicable to other fields of antiferromagnetic spintronics, such as logics, magnonics^12^ and material characterization.

## Results

### Room temperature operation of AF-MERAM

**Figure 1:**
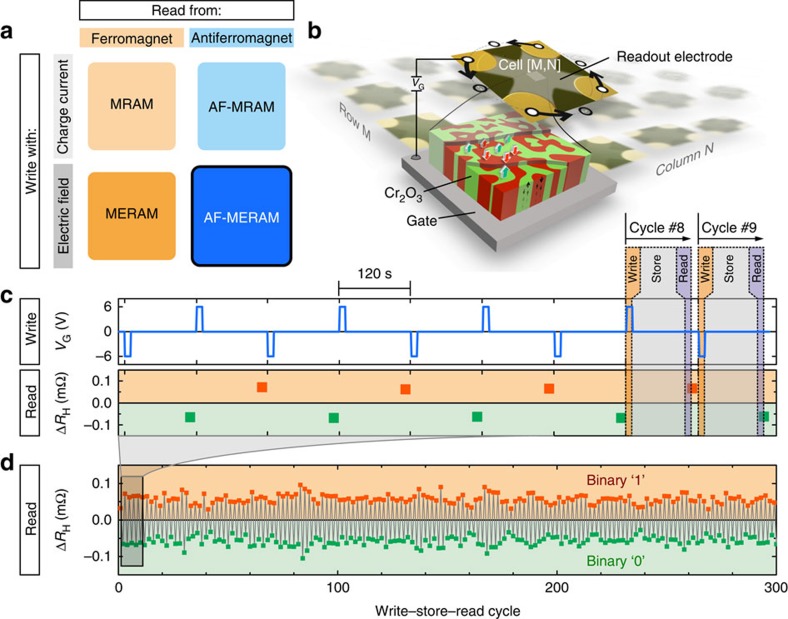
Electric field-driven manipulation of the antiferromagnetic order parameter. (**a**) Nonvolatile magnetic random access memory elements categorized according to their writing and readout interfaces. Antiferromagnetic magnetoelectric random access memory (AF-MERAM) initiates a new field of antiferromagnetic spintronics. (**b**) Sketch of one memory cell within a matrix of devices. The arrows indicate the contact permutation to obtain offset free Hall readings^10^. (**c**) Random access memory operation where binary information is written by a voltage pulse and stored in the antiferromagnetic order parameter. The magnetic state is readout at a later time after the writing stimulus is removed. (**d**) Device behavior over 300 write–store–read cycles.

To realize the memory cell, we use an epitaxial layer stack of Pt(20 nm)/α-Cr_2_O_3_(200 nm)/Pt(2.5 nm) that is prepared on Al_2_O_3_(0001) substrates. Similar stacks with α-Cr_2_O_3_ have been extensively studied in the scope of traditional MERAM elements with ferromagnetic Co layers^4^,^5^,^13^,^14^,^15^. The thicker bottom Pt film serves as the gate electrode and the thin Pt top layer is used to measure the AF order parameter all-electrically via zero-offset anomalous Hall magnetometry^10^ (hereafter zero-offset Hall). This readout approach makes use of the uncompensated boundary magnetization of α-Cr_2_O_3_(0001), which is rigidly coupled to the AF bulk and creates proximity magnetization in the Pt film^16^,^17^. An individual magnetoelectric element is obtained by patterning the top Pt layer. Figure 1c shows the protocol of an isothermal magnetoelectric switching experiment that was carried out at 19 °C in a permanent magnetic field of *H*≈+0.5 MA m^−1^ along the film normal. The test sequence mimics random access operations comprising the three essential elements of any memory cell: writing, storage and reading. One of the key technological advantages is that the memory cell operates in static magnetic fields and writing operations are triggered by the application of a voltage. No energy input is necessary during the storage times. The reproducibility of this process is demonstrated over 300 write–store–read cycles in Fig. 1d, during which the cell reveals no performance degradation.

**Figure 2:**
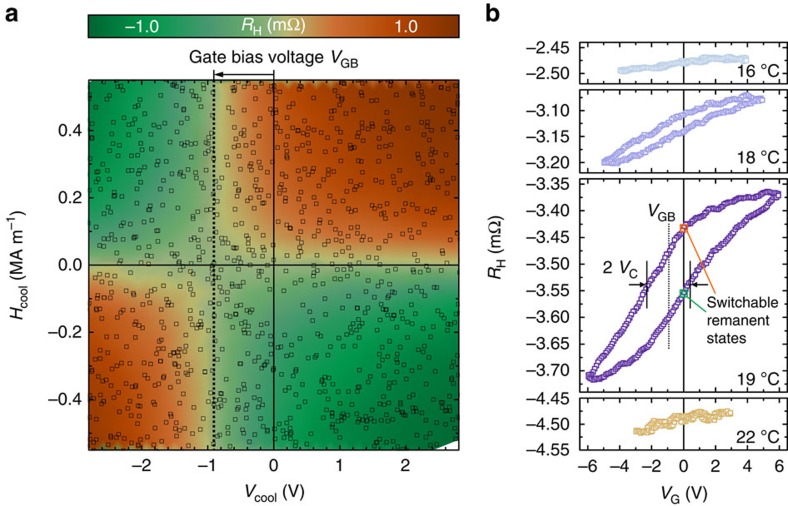
Isothermal and field-cooled magnetoelectric selection. (**a**) Map of the antiferromagnetic state selected by a range of magnetic field and gate voltage combinations during cool-down from 30 °C through the antiferromagnetic ordering temperature to the measurement temperature of 7 °C. Measurements were carried out at *H*=0 and *V*_G_=0. The squares are data points and the background color is a linear interpolation. (**b**) Readout signal corresponding to the antiferromagnetic order parameter of the cell as a function of the writing voltage *V*_G_ for several temperatures near the antiferromagnetic ordering temperature and *H*=0.5 MA m^−1^. The open hysteresis loop with coercivity *V*_C_ gives rise to switchable remanent states.

Two key material requirements must be satisfied to achieve reliable magnetoelectric reversal processes such as shown in Fig. 1. First, the order parameter has to be susceptible to the gate voltage via the linear magnetoelectric effect. Second, the cell has to exhibit thermal stability at the operation temperature, giving rise to stable remanent magnetic states. Both criteria can be directly probed in our system using the electrical writing and reading interfaces of the magnetoelectric cell. To reveal the exact influence of magnetic and electric field on the antiferromagnetic order parameter, it is mandatory to avoid the influence of magnetic anisotropy, which fixes the order parameter while below the ordering temperature, and instead carry out magnetoelectric field cooling through the ordering temperature. The map in Fig. 2a shows the resulting average antiferromagnetic order parameter in the cell after cooling from 30 to 7 °C using the indicated combination of magnetic cooling field *H*_cool_ and electric cooling field *E*_cool_=*V*_cool_/*t* (*t* denotes the AF film thickness). For large *EH* fields, the order parameter selection is consistent with that expected in α-Cr_2_O_3_ (refs 7, 13, 18, 19) due to the linear magnetoelectric effect. However, for small writing voltages that are technologically desirable, the *EH* symmetry is disturbed, giving rise to magnetic field-induced selection of the order parameter. Strikingly, the *EH* symmetry is perfectly restored when accounting for a gate bias voltage *V*_GB_, which is about −1 V for this system.

When applying a writing voltage to the cell at 19 °C, the antiferromagnetic order parameter can be switched hysteretically with a coercive gate voltage *V*_C_ of ≈1.5 V (Fig. 2b), completing the list of ingredients for the nonvolatile AF-MERAM prototype. The slightly asymmetric shape of the hysteresis loop is due to the gate voltage range being symmetric about *V*_G_=0, instead of *V*_G_=*V*_GB_. The temperature window, in which magnetoelectric writing can be carried out, is limited at higher temperatures by the collapse of antiferromagnetic order and at lower temperatures by magnetic anisotropy^15^. It should be possible to widen this writability window considerably to >100 K. The high-temperature limit can be enhanced by doping^20^,^21^, and the lower-temperature limit by applying higher writing voltages^4^,^5^ or by intentionally reducing the anisotropy via doping^20^ (Supplementary Note 1).

Table 1 contains an overview of state-of-the-art studies of magnetoelectric functionality using magnetoelectric thin films^4^,^5^,^6^,^9^,^13^ and single crystals^7^, but in both cases relying on interfacial exchange bias with a thin ferromagnetic layer. In addition, the AF-MERAM cell presented in this work is included for comparison. The metrics in the overview are the magnetoelectric film thickness *t*, the writing threshold (*VH*)_C_ and the coercive gate voltage *V*_C_. For integration in microelectronics, the latter two are of particular relevance as the circuit voltage rating depends on them.

While exchange bias has traditionally been used to probe the antiferromagnetic state of Cr_2_O_3_, this leads to strongly increased magnetoelectric coercivities, especially for thin films of Cr_2_O_3_ (refs 4, 5). When judging the writing threshold, all the exchange bias systems require very large *VH* for isothermal magnetoelectric switching of the AF order parameter in Cr_2_O_3_. In contrast, the AF-MERAM approach provides a route to reduce both the coercivity and the resulting write voltage by a factor of about 50 over exchange-biased examples (Supplementary Note 2). In addition, AF-MERAM can be readout in permanent external magnetic fields, whereas exchange-biased MERAM requires the removal of the magnetic field for readout. Thus, the example here presented opens an appealing field of AF-MERAM with ultra-low writing thresholds and superior stability and readability of the magnetic information in the presence of external magnetic fields.

## Discussion

The gate bias voltage of 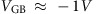 (Fig. 2) presents a key challenge for achieving ultra-low voltage threshold switching and ultra-high data stability. It has a detrimental effect on both the required writing voltage and on the data stability at zero voltage as the antiferromagnetic state develops a susceptibility to magnetic fields, even in the absence of an electric field (*V*_G_=0). We find that the gate bias voltage is a material characteristic in thin films of magnetoelectric antiferromagnets, and in the following we reveal its physical origin and derive a means to control its value.

When combining the large body of data on Cr_2_O_3_ thin-film systems^4^,^5^,^7^,^9^,^13^,^15^,^17^,^22^,^23^,^24^,^25^,^26^,^27^,^28^,^29^, a coherent picture emerges: the total magnetoelectric energy density exerting a selection pressure on the antiferromagnetic order parameter in thin-film magnetoelectric antiferromagnets is composed of three effects that act simultaneously:


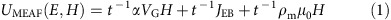


The first term describes the linear magnetoelectric effect with its coefficient *α* reported to be about 1 ps m^−1^ in Cr_2_O_3_ (refs 18, 30). This is the only desired effect in the context of MERAM devices, while the other two effects are parasitic. The second term is the influence of the exchange bias coupling strength *J*_EB_ on the antiferromagnet. While this term was typically the strongest contribution in previous studies (Supplementary Note 3), it is zero in AF-MERAM due to the lack of a ferromagnet. The last term arises from a non-zero areal magnetic moment density *ρ*_m_ within the antiferromagnet itself, which renders the material ferrimagnetic. This term, due to emergent ferrimagnetism, cannot be excluded a priori. The gate bias voltage can now be calculated from equation (1):


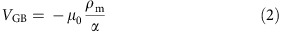


The gate bias voltage *V*_GB_ is in an intimate relation with the magnetoelectric coefficient α and the areal magnetic moment density *ρ*_m_ at the onset temperature of the thermal stability of the antiferromagnetic order. Equation (2) implies that the gate bias in magnetoelectric field cooling experiments vanishes for perfectly antiferromagnetic order (*ρ*_m_=0). Its non-zero value in our system can be used to estimate the approximate ferrimagnetic moment density at the ordering temperature of about 21 °C, yielding a value of 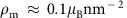. Conversely, achieving a low gate bias voltage requires that ferrimagnetism is averted.

The presence of ferrimagnetism cannot be accounted for by any intrinsic effect within the Cr_2_O_3_ antiferromagnetic film, as all magnetic moments, including boundary moments^10^,^16^,^17^, are intrinsically compensated when accounting for all boundaries (Supplementary Note 4; Supplementary Fig. 1). Therefore, extrinsic effects are necessary to break the sublattice equivalence and produce ferrimagnetism. In the following, we present an in-depth study of extrinsic thin-film phenomena and their influence on the emergent ferrimagnetism. Namely, we invoke different degrees of crystalline twinning, elastic lattice deformation, intermixing and misfit dislocation density in Cr_2_O_3_ thin films, by preparing three distinct systems with epitaxial underlayers of Al_2_O_3_(0001), Pt(111) or V_2_O_3_(0001).

**Figure 3:**
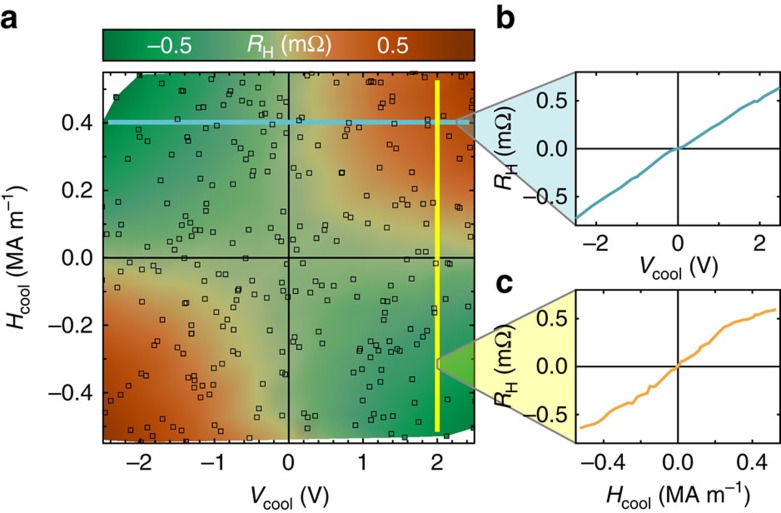
Influence of a V_2_O_3_ buffer layer on Cr_2_O_3_ magnetoelectricity. (**a**) Magnetoelectric field cooling map of the antiferromagnetic order selection for a V_2_O_3_/Cr_2_O_3_/Pt system. (**b**,**c**) Line profiles taken from the map in **a**. Only non-zero products of gate voltage and magnetic field lead to appreciable order parameter selection, whereas the individual stimuli do not.

One striking result is that the gate bias voltage, and thus the emergent ferrimagnetism, can indeed be controlled by the choice of underlayer material. In particular, when Cr_2_O_3_ thin films are grown on a V_2_O_3_ underlayer, ferrimagnetism is almost entirely eliminated. Figure 3a shows a magnetoelectric field cooling map of the V_2_O_3_-buffered system exhibiting virtually perfect *EH* symmetry. As highlighted by the indicated line profiles (Fig. 3b,c), the selection preference for a particular antiferromagnetic state vanishes when either *E*=0 or *H*=0, as expected from the pristine action of the linear magnetoelectric effect (first term in equation (1)). The possibility to completely eliminate the gate bias is highly relevant for AF-MERAM applications, as the AF state can then be switched with lower voltages (Supplementary Note 5; Supplementary Fig. 2) and is completely stable once the gate voltage returns to zero.

**Figure 4:**
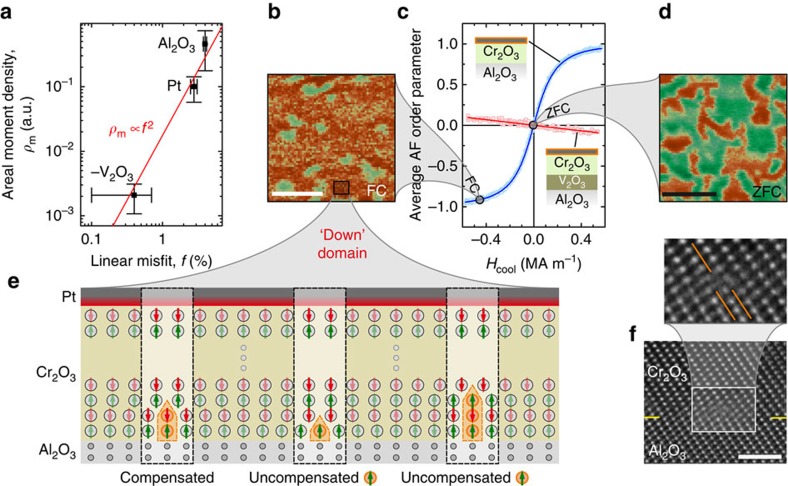
Thin-film Cr_2_O_3_ behaves ferrimagnetically. (**a**) Scaling between the measured areal magnetic moment density and the linear misfit between Cr_2_O_3_ and its underlayer. Vertical error bars represent s.e.'s based on the best fit values for the domain moment and domain areas (Table 2; Supplementary Note 7). Horizontal error bars show the maximum discrepancy of the lattice misfit values when accounting for only half the thermal expansion of one lattice. (**b**,**d**) Images of the surface magnetic stray field after field cooling (FC) and zero-field cooling (ZFC), respectively, were obtained by scanning nitrogen-vacancy microscopy (see text). Scale bars, 1 μm. (**c**) The emergent ferrimagnetism couples strongly to external magnetic fields and renders the antiferromagnetic order parameter selectable by magnetic fields much smaller than anisotropy fields. (**e**) Sketch of the effect of different misfit dislocations on the atomic populations of the two antiferromagnetic sublattices. (**f**) High-resolution TEM images of the Al_2_O_3_/Cr_2_O_3_ interface (yellow guide lines) showing complete structural coherence disrupted by occasional misfit dislocations. Scale bar, 2 nm.

To pinpoint the specific extrinsic effect that is responsible for the different degrees of emergent ferrimagnetism in Cr_2_O_3_ thin films grown on different underlayers, it is instructive to correlate the observed areal magnetic moment density and the various growth-induced effects (Table 2). The areal magnetization is determined via the slope of the dependence of the antiferromagnetic order parameter selection on the magnetic field (Fig. 4c; Supplementary Fig. 5). The gradual shape of these dependences results from a selection tendency of uniaxial antiferromagnetic domains according to their ferrimagnetism, averaged over the readout electrode area. To verify that the microscopic ordering is indeed a mixture of purely uniaxial domains, images of the surface magnetization states after zero-field cooling (Fig. 4d) and field cooling (Fig. 4b) were obtained by scanning nitrogen-vacancy (NV) magnetometry^31^,^32^,^33^. This technique measures the stray magnetic field 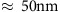 above the sample surface, clearly indicating the equal presence of up- and down-domains in the zero-field-cooled state. In contrast, field cooling predominantly selects one of the two domain orientations that allows to calculate the degree of ferrimagnetism in the films.

It should also be noted that measuring the gate bias voltage *V*_GB_ (Equation (2)) provides a second route to quantify the areal magnetization *ρ*_m_ absolutely, which is however restricted to conducting underlayers and suffers from the uncertainty of the value of *α*. Therefore, magnetization values determined via the gate bias will not be used for the comparison of the different underlayer materials.

Based on these data, we conclude that elastic film strain, twinning and cation intermixing in epitaxial Cr_2_O_3_ films cannot account for the observed degree of ferrimagnetism, as none of these properties are correlated to the areal magnetization (Table 2). Instead, the results suggest that the lattice mismatch is the cause of the emergent ferrimagnetism.

The scaling relationship between the measured areal magnetic moment density and the linear lattice misfit between Cr_2_O_3_ and its underlayer is shown in Fig. 4a. When taking into account the data of the three investigated systems, we find that the data align tightly to a quadratic scaling relation (red line). Such a relationship hints at the number of the misfit dislocations per area being the key property determining the areal ferrimagnetic moment density. This result leads to a picture in which the population of the two antiferromagnetic sublattices is unbalanced by the presence of misfit dislocations.

Such dislocations arise due to the heteroepitaxial film growth (Fig. 4f) as the dominant defect type of the otherwise highly coherent interface and appear within the first atomic layers of the Cr_2_O_3_ film as evidenced by positron annihilation spectroscopy (Supplementary Note 8; Supplementary Fig. 7; Supplementary Table 1). As sketched for the case of compressive misfit in Fig. 4e, the dislocations (orange boxes) can contain unequal populations of spin ‘up' and spin ‘down' atoms if the dislocation terminates after an odd number of atomic layers. These surplus spins are all aligned within one domain due to the layered sublattice structure in α-Cr_2_O_3_(0001). While the magnetic moment of atoms near dislocations might be different from atoms in the relaxed lattice, this picture serves to illustrate that misfit dislocations do indeed unbalance the atomic populations in each of the two sublattices.

Remarkably, the lattice misfit not only correlates with the magnitude of the emergent magnetization, but also with its sign with respect to the antiferromagnetic order parameter. This sign change of the ferrimagnetic behavior in the case of tensile misfit for the V_2_O_3_-buffered sample emerges naturally from the previously introduced picture. Tensile misfit results in atoms being skipped from the bottom boundary sublattice instead of atoms being added. Therefore, tensile misfit results in the top boundary magnetization being aligned along the cooling field, while compressive misfit results in the top boundary magnetization being aligned opposite to the magnetic cooling field.

To quantify ferrimagnetism, we investigate the magnetic field-induced antiferromagnetic order parameter selection with no electric field applied (Fig. 4c), which is influenced exclusively by the last term of equation (1). The Al_2_O_3_ buffered films are clearly more susceptible to the magnetic field than the V_2_O_3_-buffered films, which is in line with the substantially larger lattice misfit of the former over the latter. Moreover, a magnetic field of the same sign selects opposite antiferromagnetic states in the two systems, which corresponds to the opposite sign of the lattice misfit.

In conclusion, we demonstrated reliable room temperature magnetoelectric random access memory cells based on a new scheme that relies purely on antiferromagnetic components and does not require a ferromagnet for readout. This AF-MERAM provides substantially reduced writing thresholds over conventional MERAM prototypes, enabling further improvements in the energy efficiency of nonvolatile solid-state memory and logics. Since a permanent magnetic writing field does not interfere with readout in AF-MERAM, this new approach extends voltage driven writing to magnetoelectric antiferromagnets such as Cr_2_O_3_, whereas such functionality has previously been feasible only in multiferroic antiferromagnets such as BiFeO_3_ (Table 1). It should be noted that the advantages of omitting the ferromagnet from MERAM cells likewise apply to multiferroic antiferromagnets, opening an appealing field of AF-MERAM with ultra-low writing thresholds and superior stability of the magnetic order parameter. The concept also provides an important new building block for the emerging field of antiferromagnetic spintronics. While we did not investigate the speed of the actual writing process, first prototypes of conventional MERAM could be switched within a few tens of nanosecond^5^.

We use thin films of magnetoelectric antiferromagnetic Cr_2_O_3_ as the core material and find that this material becomes ferrimagnetic when grown as epitaxial thin films. Emergent ferrimagnetism in thin films of magnetoelectric antiferromagnets can be desirable^34^. For the application to purely antiferromagnetic magnetoelectric elements, however, ferrimagnetism should be minimized. Through an in-depth structural characterization, we find that the observed degree of ferrimagnetism is correlated with the square of the linear lattice misfit between Cr_2_O_3_ and its underlayer. This finding provides both a fundamental mechanism for the phenomenon of emergent ferrimagnetism and suggests a readily available tuning knob to enhance or eliminate the magnetic field coupling of magnetoelectric antiferromagnets.

## Methods

### Sample preparation

Oxide films were grown by reactive evaporation of the base metal in high vacuum onto *c*-cut sapphire substrates (Crystec GmbH) heated to 700 °C initially and to 500 °C after the first few monolayers. The background gas used was molecular oxygen at a partial pressure of 10^−5^ mbar. Chromium was evaporated from a Knudsen cell, vanadium was evaporated from a block target using an electron-beam and platinum was sputtered from a d.c. magnetron source. Deposition of the oxides was carried out using rates of about 0.4 Å s^−1^ and was monitored *in situ* by reflection high-energy electron diffraction. Oxide layers were subjected to a vacuum annealing process at 750 °C and residual pressure of 10^−7^ mbar directly after growth. The thin Pt top layers were deposited at lower temperatures of ≈100 °C using a higher rate of 1.0 Å s^−1^ to maintain layer continuity. Hall crosses were patterned from the top Pt layers, by SF_6_ reactive ion etching around a photoresist mask.

### Transport experiments

Transport was measured using zero-offset Hall^10^. Typical current amplitudes were on the order of 500 μA. RAM operation was carried out in a permanent magnetic background field of 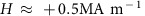 along the film normal.

To obtain the average AF order parameter dependence on the magnetic cooling field, the data of the spontaneous Hall signal after cooling under a range of field values were fitted by an expression that provides the normalization and the absolute magnetic moment distribution of individual domain pieces within the Cr_2_O_3_ films (Supplementary Note 7). The relative domain sizes of films with different buffer layers were determined using zero-offset Hall by evaluating the statistics of the domain selection within the finite-size Hall crosses (Supplementary Note 7).

The structural properties of the Cr_2_O_3_ films on different buffer layers were characterized by x-ray diffraction and channeling contrast scanning electron microscopy as shown in detail in (Supplementary Note 6).

### NV magnetic microscopy

Scanning NV magnetometry was performed with a tip fabricated from single-crystal, <100>-oriented diamond that was implanted with ^14^N ions at 6 keV, and annealed at 800 °C to form NV centres^35^.An external field of 2.2 kA m^−1^ was applied along the NV axis (diamond <111> crystal direction) to induce Zeeman splitting of the NV electronic ground-state spin. A microwave driving field was then locked to the spin transition at ≈2.864 GHz to track the additional Zeeman shift due to the stray field of the magnetic film surface^36^. Magnetic field values for each pixel were obtained by averaging the microwave lock frequency for 7 s.

### Data availability

The data underlying the present work are available upon request from the corresponding authors.

## Additional information

**How to cite this article:** Kosub, T. *et al*. Purely antiferromagnetic magnetoelectric random access memory. *Nat. Commun.* **8,** 13985 doi: 10.1038/ncomms13985 (2017).

**Publisher's note:** Springer Nature remains neutral with regard to jurisdictional claims in published maps and institutional affiliations.

## Supplementary Material

Supplementary Information Supplementary Figures, Supplementary Table, Supplementary Notes and Supplementary References

Peer Review File

## Figures and Tables

**Table 1:** Performance chart of MERAM systems.

**Study**	***t*** **(μm)**	**(*****VH*****)**_**C**_ **(MW** **m**^**−1**^**)**	**V**_**C**_ **(V)**	**Magnetic field**
Exchange bias reversal*, Cr_2_O_3_/Co/Pd (ref. 7)	1,000	240	450†	Writing pulse
Exchange bias reversal*, Cr_2_O_3_/Co/Pt (ref. 5)	0.2	40	56†	Writing pulse
Exchange bias reversal*, Cr_2_O_3_/Co/Pt (ref. 4)	0.5	48	105†	Writing pulse
Magnetization switching, BiFeO_3_/CoFe (ref. 6)	0.1	—	4	Must be ≈0 for readout
Magnetization switching, Cr_2_O_3_/Pt (present work)	0.2	0.75	1.5	Permanent

Overview of state-of-the-art isothermal magnetoelectric switching studies using either the linear magnetoelectric effect in Cr_2_O_3_ or the multiferroic coupling in BiFeO_3_. The value (*VH*)_C_ gives the magnetoelectric writing threshold (product of magnetic field and voltage). The writing voltage *V*_C_ allows to qualitatively compare Cr_2_O_3_-based systems and BiFeO_3_ systems in terms of the voltage at which the magnetization state switches.

^*^Application of the writing voltage does not switch the ferromagnetic Co, but only the antiferromagnetic Cr_2_O_3_, implying that the magnetic field must be removed for readout from the ferromagnet.

^†^For comparability, the writing voltages are calculated for a magnetic field of *H*_write_=0.5 MA m^−1^ as was used in the present study. The actual used writing voltages in these studies are similar to the normalized values, as the magnetic fields were also similar.

**Table 2:** Influence of different underlayers on structural and ferrimagnetic properties of Cr_2_O_3_ thin films.

**Underlayer material**	**Twinning ratio (%)**	***c*** **axis compression (%)**	**Expected miscibility**	**Linear misfit (%)**	**Areal magnetization** ***ρ***_**m**_ **(a.u.)**
Al_2_O_3_	≈2	0.0	weak	+4.0	+0.455±0.28
Pt	≈50	0.18	none	+2.8	+0.100±0.043
V_2_O_3_	≈2	0.30	strong	−0.5	−0.0021±0.001

The values for the structural properties are derived in detail in the (Supplementary Note 6; Supplementary Fig. 3; Supplementary Fig. 4). The ferrimagnetic moment density values are relative values obtained by zero-offset Hall (Supplementary Note 7; Supplementary Fig. 5; Supplementary Fig. 6). They are normalized to the approximate value for Pt/Cr_2_O_3_/Pt obtained via the gate bias voltage as of equation (2).

## References

[b1] Yang J. J., Strukov D. B. & Stewart D. R. Memristive devices for computing. Nat. Nanotechnol. 8, 13–24 (2013). 23269430 10.1038/nnano.2012.240

[b2] Jungwirth T., Marti X., Wadley P. & Wunderlich J. Antiferromagnetic spintronics. Nat. Nanotechno. 11, 231–241 (2016). 10.1038/nnano.2016.18 26936817

[b3] Wadley P. . Electrical switching of an antiferromagnet. Science 351, 587–590 (2016). 26841431 10.1126/science.aab1031

[b4] Ashida T. . Isothermal electric switching of magnetization in Cr_2_O_3_/Co thin film system. Appl. Phys. Lett. 106, 132407 (2015).

[b5] Toyoki K. . Magnetoelectric switching of perpendicular exchange bias in Pt/Co/α-Cr_2_O_3_/Pt stacked films. Appl. Phys. Lett. 106, 162404 (2015).

[b6] Heron J. . Deterministic switching of ferromagnetism at room temperature using an electric field. Nature 516, 370–373 (2014). 25519134 10.1038/nature14004

[b7] He X. . Robust isothermal electric control of exchange bias at room temperature. Nat. Mater. 9, 579–585 (2010). 20562879 10.1038/nmat2785

[b8] Matsukura F., Tokura Y. & Ohno H. Control of magnetism by electric fields. Nat. Nanotechnol. 10, 209–220 (2015). 25740132 10.1038/nnano.2015.22

[b9] Toyoki K. . Switching of perpendicular exchange bias in Pt/Co/Pt/α-Cr_2_O_3_/Pt layered structure using magneto-electric effect. J. Appl. Phys. 117, 17D902 (2015).

[b10] Kosub T., Kopte M., Radu F., Schmidt O. G. & Makarov D. All-electric access to the magnetic-field-invariant magnetization of antiferromagnets. Phys. Rev. Lett. 115, 097201 (2015). 26371676 10.1103/PhysRevLett.115.097201

[b11] Marti X. . Room-temperature antiferromagnetic memory resistor. Nat. Mater. 13, 367–374 (2014). 24464243 10.1038/nmat3861

[b12] Rovillain P. . Electric-field control of spin waves at room temperature in multiferroic BiFeO_3_. Nat. Mater. 9, 975–979 (2010). 21076416 10.1038/nmat2899

[b13] Ashida T. . Observation of magnetoelectric effect in Cr_2_O_3_/Pt/Co thin film system. Appl. Phys. Lett. 104, 152409 (2014).

[b14] Iwata N., Kuroda T. & Yamamoto H. Mechanism of growth of Cr_2_O_3_ thin films on (1102), (1120) and (0001) surfaces of sapphire substrates by direct current radio frequency magnetron sputtering. Jpn. J. Appl. Phys. 51, 11PG12 (2012).

[b15] Fallarino L., Berger A. & Binek C. Magnetic field induced switching of the antiferromagnetic order parameter in thin films of magnetoelectric chromia. Phys. Rev. B 91, 054414 (2015).

[b16] Belashchenko K. D. Equilibrium magnetization at the boundary of a magnetoelectric antiferromagnet. Phys. Rev. Lett. 105, 147204 (2010). 21230865 10.1103/PhysRevLett.105.147204

[b17] Wu N. . Imaging and control of surface magnetization domains in a magnetoelectric antiferromagnet. Phys. Rev. Lett. 106, 087202 (2011). 21405596 10.1103/PhysRevLett.106.087202

[b18] Fiebig M. Revival of the magnetoelectric effect. J. Phys. D Appl. Phys. 38, R123 (2005).

[b19] Dzyaloshinskii I. E. On the magneto-electrical effect in antiferromagents. J. Exp. Theor. Phys. 37, 881–882 (1959).

[b20] Mu S., Wysocki A. L. & Belashchenko K. D. Effect of substitutional doping on the Néel temperature of Cr_2_O_3_. Phys. Rev. B 87, 054435 (2013).

[b21] Street M. . Increasing the Néel temperature of magnetoelectric chromia for voltage-controlled spintronics. Appl. Phys. Lett. 104, 222402 (2014).

[b22] Shiratsuchi Y., Fujita T., Oikawa H., Noutomi H. & Nakatani R. High perpendicular exchange bias with a unique temperature dependence in Pt/Co/α-Cr_2_O_3_(0001) thin films. Appl. Phys. Exp. 3, 113001 (2010).

[b23] Shiratsuchi Y. . High-Temperature regeneration of perpendicular exchange bias in a Pt/Co/Pt/α-Cr_2_O_3_/Pt thin film system. Appl. Phys. Exp. 6, 123004 (2013).

[b24] Fallarino L., Berger A. & Binek C. Giant temperature dependence of the spin reversal field in magnetoelectric chromia. Appl. Phys. Lett. 104, 022403 (2014).

[b25] Nozaki T. . Positive exchange bias observed in Pt-inserted Cr_2_O_3_/Co exchange coupled bilayers. Appl. Phys. Lett. 105, 212406 (2014).

[b26] Shiratsuchi Y., Nakatani T., Kawahara S. & Nakatani R. Magnetic coupling at interface of ultrathin Co film and antiferromagnetic Cr_2_O_3_(0001) film. J. Appl. Phys. 106, 033903 (2009).

[b27] Lim S.-H. . Exchange bias in thin-film (Co/Pt)_3_/Cr_2_O_3_ multilayers. J. Magn. Magn. Mater. 321, 1955–1958 (2009).

[b28] Sahoo S. & Binek C. Piezomagnetism in epitaxial Cr_2_O_3_ thin films and spintronic applications. Phil. Mag. Lett. 87, 259–268 (2007).

[b29] Shiratsuchi Y. . Detection and *in situ* switching of unreversed interfacial antiferromagnetic spins in a perpendicular-exchange-biased system. Phys. Rev. Lett. 109, 077202 (2012). 23006398 10.1103/PhysRevLett.109.077202

[b30] Folen V. J., Rado G. T. & Stalder E. W. Anisotropy of the magnetoelectric effect in Cr_2_O_3_. Phys. Rev. Lett. 6, 607–608 (1961).

[b31] Balasubramanian G. . Nanoscale imaging magnetometry with diamond spins under ambient conditions. Nature 455, 648–651 (2008). 18833276 10.1038/nature07278

[b32] Maletinsky P. . A robust scanning diamond sensor for nanoscale imaging with single nitrogen-vacancy centres. Nat. Nanotechnol. 7, 320–324 (2012). 22504708 10.1038/nnano.2012.50

[b33] Taylor J. . High-sensitivity diamond magnetometer with nanoscale resolution. Nat. Phys. 4, 810–816 (2008).

[b34] Halley D. . Size-induced enhanced magnetoelectric effect and multiferroicity in chromium oxide nanoclusters. Nat. Commun. 5, 3167 (2014). 24452260 10.1038/ncomms4167

[b35] Appel P. . Fabrication of all diamond scanning probes for nanoscale magnetometry. Rev. Sci. Instrum. 87, 063703 (2016). 27370455 10.1063/1.4952953

[b36] Schoenfeld R. S. & Harneit W. Real time magnetic field sensing and imaging using a single spin in diamond. Phys. Rev. Lett. 106, 030802 (2011). 21405264 10.1103/PhysRevLett.106.030802

