# Supplementary material for: Purely antiferromagnetic magnetoelectric random access memory
Source: Nat Commun. 2017 Jan 3;8:13985. doi: 10.1038/ncomms13985 (PMC5216083; doi:10.1038/ncomms13985)
Supplement: Supplementary Information — Supplementary Figures, Supplementary Table, Supplementary Notes and Supplementary References [file ncomms13985-s1.pdf]

Supplementary Information for

Purely Antiferromagnetic Magnetoelectric Random Access Memory

Tobias Kosub[1,2], Martin Kopte[1,2], Ruben Hühne[3], Patrick Appel[4], Brendan Shields[4], Patrick Maletinsky[4], René Hübner[2], Maciej Oskar Liedke[5], Jürgen Fassbender[2], Oliver G. Schmidt[1], Denys Makarov[1,2]

[1]Institute for Integrative Nanosciences, Institute for Solid State and Materials Research (IFW Dresden e.V.), 01069 Dresden, Germany. [2]Helmholtz-Zentrum Dresden-Rossendorf e.V., Institute of Ion Beam Physics and Materials Research, 01328 Dresden, Germany. [3]Institute for Metallic Materials, Institute for Solid State and Materials Research (IFW Dresden e.V.), 01069 Dresden. [4]Department of Physics, University of Basel, 4056 Basel, Switzerland. [5]Helmholtz-Zentrum Dresden-Rossendorf e.V., Institute of Radiation Physics, 01328 Dresden, Germany. Correspondence and requests should be addressed to T.K. (email: t.kosub@hzdr.de) and D.M. (email: d.makarov@hzdr.de).

Supplementary Notes

1. Effects of substitutional doping on the magnetic properties of Cr_2O_3

The Néel temperature of pristine Cr_2O_3 of about 307 K is too low to be worthwhile for commercial room-temperature applications. This issue has previously been investigated both theoretically[1] and the predictions were later confirmed in experiment qualitatively and quantitatively[2]. Namely, the Néel temperature can be enhanced to roughly 400 K by about 3 % substitutional anion doping of boron for oxygen .Therefore, we consider the predictions regarding intentional cation doping obtained by the same calculations as highly relevant.

As shown in Ref. [1] different dopants display differences in the details of their substitutional effect. Some dopants (Ni, Co) affect nearest neighbor exchange more severely than long range exchange. Some dopants such as Mn or Fe are predicted to increase the average sublattice magnetization while the other dopants are predicted to reduce it. One should also notice, that α-Fe_2O_3 and α-Ti_2O_3 are themselves corundum structure antiferromagnets, but with different antiferromagnetic order. In particular, Fe_2O_3 possesses a Néel temperature of 950 K, but no linear magnetoelectric effect in its pristine form.

All of these facts lead us to the conclusion, that the substitutional doping of Cr_2O_3 is a complex endeavor that can yield various effects like the demonstrated increase of the Néel temperature or the change of the antiferromagnetic order type. Combining different dopants is therefore likely to address both the Néel temperature and the magnetic anisotropy, possibly to different or even inverse extents.

2. Calculation of the writing threshold reduction factor of AF-MERAM compared to MERAM

Although comparisons between results obtained for thin film system prepared by different groups in different chambers and different fabrications habits are often complicated, the situation is less ambiguous in the case of Cr_2O_3-based thin film MERAM prototypes. All of the successful demonstrations of these prototypes are based on the same material system, which yields consistent results throughout several groups. This system is based on (0001) cut Sapphire single crystal substrates, a roughly 20 nm thick sputtered Pt gate layer, a roughly 200 nm thick Cr_2O_3 layer prepared at about 600°C and a metallic sensing layer sputtered at room temperature. Throughout the last decade, this last sensing layer has been very thoroughly

characterized[3–13]. One of the key conclusions is that the stronger the exchange bias between Cr_2O_3 and a ferromagnetic Co sensing layer, the easier it is to read out the system (due to the larger shift of the ferromagnetic hysteresis loop) but the harder it is to write the antiferromagnet via the magnetoelectric effect[13]. By finely tuning the exchange bias strength via intentional decoupling of Cr_2O_3 and Co, several groups eventually managed to achieve both writability of Cr_2O_3 and stable exchange bias at low temperatures. The few works which demonstrate this functionality[7,9,12] are all included as performance references in our manuscript and are best-case scenarios selected from a much wider body of research work. Many articles published within the last decade, but also internal work done by us, showed reliable exchange bias between Cr_2O_3 and Co, reliable isolation of the gate electrode but no writability by the magnetoelectric effect because the necessary writing voltages were beyond the dielectric breakdown strength of Cr_2O_3[4].

On the other hand, magnetoelectric switching of pristine Cr_2O_3 without an attached ferromagnet has been performed since the discovery of the linear magnetoelectric effect in the early 1960ies. Many of these experiments yielded even lower values of the EH product than reported in the current manuscript as necessary to switch the AF order parameter in crystal samples[14] or to field cool to single domain states in thin film sample[15].

Therefore, it is very likely that by selecting the best-case writing thresholds for conventional MERAM and comparing these figures to the intermediate plain Cr_2O_3 writing threshold obtained by our AF-MERAM prototype, we are in fact underestimating the reduction factor of the writing thresholds that is achieved by removing the ferromagnetic layer from a thin film MERAM system. For structurally entirely comparable MERAM and AF-MERAM systems, the reduction factor of the writing threshold could thus be even higher than 50-fold compared to the ferromagnet-containing counterparts.

A vague estimation of the reduction factor that could be achieved by removing the ferromagnet from optimized traditional MERAM system, can be learnt when comparing the energy contributions to the domain selection in magnetoelectric antiferromagnets [Supplementary Note 3]. These latter consideration yield an estimated reduction factor of the writing threshold of AF-MERAM compared to traditional MERAM on the order of 1000.

3. Energy contributions to antiferromagnetic domain selection in magnetoelectric antiferromagnets

To assess the relative importance of the three contributions, exemplary values for the film thickness, the electric and magnetic fields of $t = 100\,\mathrm{nm}$, $V_\mathrm{G} = 1\,\mathrm{V}$ and $H = 0.1\,\mathrm{MA\,m^{-1}}$ will be

used. These particular values represent moderate quantities that can be routinely achieved in technological applications.

The selection pressure due to the linear magnetoelectric effect [first term in Eq. (1)] is on the order of $1\ \mathrm{Pa}$ for the magnetoelectric coefficient of Cr_2O_3 of about $\alpha \approx 1\ \mathrm{ps\ m^{-1}}$[16,17]. The second term describes the interfacial exchange bias coupling energy which depends on the relative alignment between the ferromagnetic magnetization and the AF order parameter. In existing works, the coupling was usually collinear along the film normal. The coupling constant[13] is about $J_{\mathrm{EB}} = 0.1\ \mathrm{mJ\ m^{-2}}$. This effect generates of a selection pressure of about $1000\ \mathrm{Pa}$ for the assumed parameters. The last term is caused by the Zeeman energy which arises as a consequence of non-zero areal magnetic moment density ρ_m in thin films of magnetoelectric antiferromagnet. Such magnetization was attributed to surface effects and was found to be on the order of $\rho_m = 0.1\ \mu_{\mathrm{B}}\ \mathrm{nm^{-2}}$ for similar systems[18]. This value can be extracted when assuming a signal level of $10^{-8}\ \mathrm{emu}$ and a sample area of $0.1\ \mathrm{cm^2}$ as are typical for the device used in Ref. [18]. Calculating the areal magnetization via the gate bias voltage as done in the main text, yields a similar magnitude. With the aforementioned parameters this contribution is also on the order of $1\ \mathrm{Pa}$.

As a result, the exchange bias contribution far outweighs the other contributions at a magnitude of about $1000\ \mathrm{Pa}$. Consequently, the writability of information via the linear magnetoelectric effect [first contribution in Eq. (1)] is severely compromised if exchange bias coupling is present.

4. Hall measurements of both surfaces of the Cr_2O_3 thin film

Supplementary Figure 1 shows magnetic hysteresis loops obtained by zero-offset Hall[19] of Cr_2O_3/Pt top and bottom interfaces, respectively. The high temperature paramagnetic curves show a negative slope with the magnetic field implying that the sign of the anomalous Hall signal in Pt is opposite to the sign of the boundary magnetization in Cr_2O_3. The reason for that is that either the anomalous Hall coefficient of Pt or the proximity magnetization in Pt with respect to Cr is negative. Magnetic field cooling in a positive field of $H_{\mathrm{cool}} = 0.5\ \mathrm{MA\ m^{-1}}$ down to 9°C is then used to induce an almost saturated AF state in the Cr_2O_3 film exploiting the weak ferrimagnetism. Such a domain state is expected to display ferromagnetic surface terminations with different signs of the magnetization at the two interfaces. As a result, the observed Hall signals of the two boundaries show opposite sign and almost identical magnitudes when considering the different thicknesses of the Pt layer. When expressed in terms of the Hall resistivities $\rho_{\mathrm{H}} = R_{\mathrm{H}} t_{\mathrm{Pt}}$, one obtains $+2.5\ \mathrm{p\Omega m}$ for the top surface and $-3\ \mathrm{p\Omega m}$ for the bottom

surface. The two readings can be judged to be of similar magnitude, but as two different samples are measured, a quantitative comparison would be ambiguous.

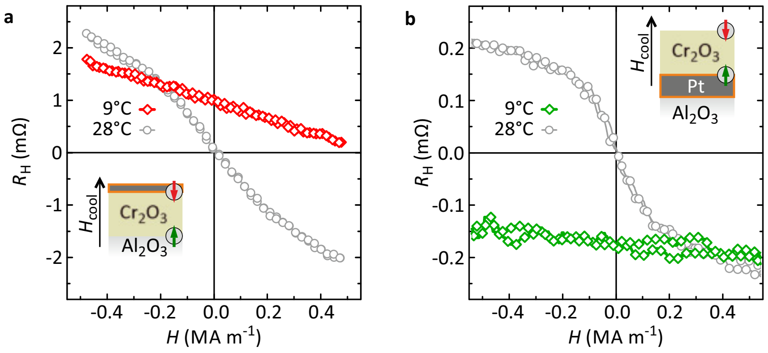

Supplementary Figure 1 | Zero-offset Hall measurements on both surfaces of the Cr_2O_3 film. a, Hall signal taken at the top surface becomes offset positively for cooling in a positive magnetic field. **b,** Hall signal taken at the bottom surface becomes offset negatively for cooling in a positive magnetic field.

These measurements confirm that the boundary magnetization at the Cr_2O_3(0001) top surface is approximately compensated by the antiparallelly aligned magnetization at the bottom surface. The ferromagnetic boundary layers thus do not conflict with perfect antiferromagnetism of Cr_2O_3 thin films or magnetoelectric antiferromagnet films in general. As a result, the areal magnetization at one boundary is expected to significantly larger than the integral ferrimagnetic moment density estimated by SQUID[18] or by the gate bias voltage [main text]. This further corroborates that the boundary magnetization is indeed largely compensated when taking the full thickness of the Cr_2O_3 film into account.

However, one can infer that the sublattice containing the bottom interface, has a larger magnetic moment, since the bottom interface magnetization aligns with the magnetic field, while the top interface is consistently aligned opposite to the magnetic field. As a result, the average AF order parameter of the Cr_2O_3 thin film becomes susceptible to medium strength magnetic fields of less than 1 MA m^{-1}.

5. Isothermal magnetoelectric switching in the V_2O_3 gated sample

V_2O_3 offers interesting possibilities as a gate material for Cr_2O_3-based MERAM. It allows to avert twinning as shown in the main text, while providing a gate electrode. Its ultra-low lattice mismatch guarantees negligible ferrimagnetism in the magnetoelectric layer. **Supplementary Figure 2** shows a room temperature hysteresis loop of the AF order parameter in dependence of the gate voltage extending to 2 V. Despite the low applied gate voltage, the hysteresis

opening is discernible. The shown curve is an average of 30 individual hysteresis loops, which leaves an uncertainty of about $5\ \mu\Omega$ in the individual data points. When comparing the upper and lower branches as wholes, the mean value of the upper branch is higher than that of the lower branch by $\Delta\langle R_\mathrm{H}\rangle = (9.97 \pm 0.53)\ \mu\Omega$. Therefore, the hysteresis loop is clearly open with a significance of $18.9\ \sigma$.

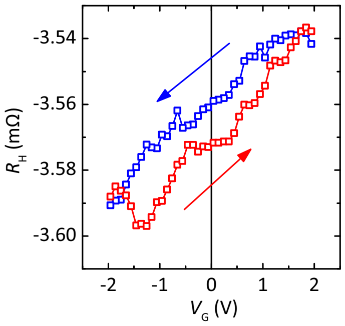

Supplementary Figure 2 | Isothermal magnetoelectric switching of Cr_2O_3 on V_2O_3. The hysteresis loop was obtained at 23°C while a permanent magnetic field of $H_\mathrm{perm} = 0.5\ \mathrm{MA\ m^{-1}}$ was applied.

When comparing the behavior of the V_2O_3 gated cell in magnetoelectric field cooling [main text] and isothermal switching to similar tests carried out for the Pt gated cells in the main text, some conclusions can be drawn. The lower lattice mismatch provided by V_2O_3 reliably suppresses the gate bias. On the other hand, the magnetoelectric susceptibility of Cr_2O_3 appears to be reduced when grown on V_2O_3. The latter could be a result of doping due to cation exchange, which is a spontaneous process at high temperatures due to the excellent miscibility of the two materials. Optimized deposition conditions can be employed to alleviate this issue.

6. Structural investigation of the Cr_2O_3 films on different underlayers

The in-plane and out-of-plane crystalline order of epitaxial thin films can be efficiently probed by a 2-dimensional reciprocal space map, if one of the crystalline axes is selected as the in-plane direction. Through varying the incident and scattering angles, one obtains an overview such as that in **Supplementary Figure 3**. The maps are all aligned in the 3-dimensional reciprocal space by the $1\ 0\ 10$ reflection of the Al_2O_3 substrate and thus one finds this reflection in all the maps. All the present reflections are identified in panel c. As Al_2O_3 is a single-crystalline substrate, its properties are highly consistent and the peak intensity is observed at the lattice position of relaxed Al_2O_3. The second reflection present in all the maps is that of Cr_2O_3. The appearance of reflections not belonging to the substrate is a clear indication that Cr_2O_3 is indeed

crystalline and its position in reciprocal space clearly identifies it as a $1\ 0\ 10$ reflection akin to the substrate reflection. To confirm the epitaxial relationship unambiguously, the in-plane symmetry can be assessed by probing the reciprocal space ring through the reflection and concentric about the $[0\ 0\ 1]$ axis. The Cr_2O_3 and V_2O_3 films grown on Al_2O_3 substrates cannot maintain the threefold symmetry [**Supplementary Figure 3**(d-f)] entirely, which is caused by the formation of grains with a second in-plane orientation during film growth. The preponderance of these grains varies strongly between the different systems. We will refer to those grains as twin grains as they have the same crystallographic orientation as rotational twins. While all oxide systems contain only about 2% such grains, Cr_2O_3 films on Pt have no preference in the selection of the twin type.

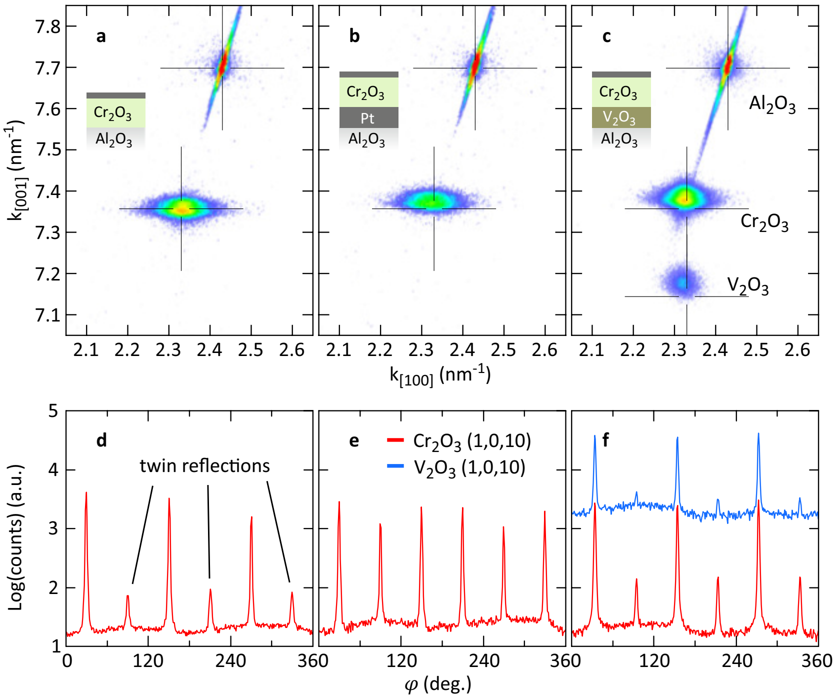

Supplementary Figure 3 | Structure characterization by X-ray diffraction. a-**c**, 2-dimensional reciprocal space maps aligned with the $[1\ 0\ 0]$ substrate axis and spanning the range around the $1\ 0\ 10$ reciprocal corundum lattice point. Black crosshairs indicate the relaxed room temperature lattice position of the three corundum oxides. **d**-**f**, Corresponding φ scans through the Cr_2O_3 (red curve) and the V_2O_3 lattice points (blue curve), which reveal the present in-plane rotational symmetry.

The in-plane lattice misfit between Cr_2O_3 and Al_2O_3 is substantial at 4.0 %. It is remarkable that the position of the Cr_2O_3 reflection exactly matches its relaxed bulk value, which is overlaid as a black cross, when Al_2O_3 is used as a substrate. This implies that the misfit strain of Cr_2O_3 on Al_2O_3 is fully relaxed by misfit dislocations. As no appreciable signature of unrelaxed Cr_2O_3 is

evident, the unrelaxed Cr_2O_3 volume must be negligible and the dislocations must be situated close to the Al_2O_3/Cr_2O_3 interface. The misfit leads to an average spacing of misfit lines of 25 Cr_2O_3 unit cells or 10.7 nm. At the same time, the φ scan of the reciprocal lattice ring [**Supplementary Figure 3**(d)] shows that the 3-dimensional symmetry is almost perfectly transferred to the Cr_2O_3 layer, apart from a 2 % minority area where crystals with a 60° in-plane rotation are present. These twins give rise to the smaller intermediate reflections in the φ scan. These data indicate that the Cr_2O_3 layer directly on Al_2O_3 obtains structural properties close to that of a single crystal although the lower interface region is substantially disturbed.

Similar measurements for the $Al_2O_3/Pt/Cr_2O_3$ system are shown in **Supplementary Figure 3**(b). In contrast to the Al_2O_3/Cr_2O_3 system, the Cr_2O_3 layers on the Pt buffer are found in a non-relaxed state after annealing and cooling down. Strain relaxation is a thermodynamically beneficial process which is limited by its slow kinetics at lower temperatures. Therefore, the maximally relaxed state is expected at the highest temperature experienced by the sample. The fully relaxed state observed in the Al_2O_3/Cr_2O_3 system implies that the annealing process is sufficient to obtain relaxed Cr_2O_3 at the annealing temperature.

The observed strain in the $Al_2O_3/Pt/Cr_2O_3$ system is developed during the cool-down to room temperature. Such thermal strains regularly arise when layers with different thermal expansion coefficients are stacked. The thermal expansion of Al_2O_3 between room temperature and 750°C is about 0.47 %[20]. That of Cr_2O_3 is expected to be similar, as Al_2O_3/Cr_2O_3 cools free of strain. Pt displays a larger thermal expansion of about 0.75 %[21]. When cooling the layer stack to room temperature, the expansion differences of the layers cannot be relaxed completely because the thermal energy of the cooling system is insufficient to invoke pronounced atomic rearrangements of the highly relaxed system. Instead, the layers retain an elastic strain if their thermal expansion ratios do not match. Due to the higher modulus and volume of the Cr_2O_3 layer compared to the Pt layer, the differential strain of the Pt/Cr_2O_3 system will be almost exclusively stored in the Pt lattice. Additionally, the complete Pt/Cr_2O_3 bilayer becomes tensely strained due to the differential Al_2O_3/Pt strain of about 0.25 %. As a result, the Cr_2O_3 layer with the Pt seed layer retains an elastic c-axis compression of about 0.18 % at room temperature.

The presence of this elastic deformation indicates that the lattice misfit at the annealing temperature is a better indicator for the estimation of the misfit location density in the Cr_2O_3 films. This lattice mismatch between Pt and Cr_2O_3 is approximately 2.8 % giving rise to misfit lines every 14.9 nm. Thus, the Pt buffer reduces the linear density of misfit locations by about one third and the areal density by about one half. On the other hand, the Cr_2O_3 layer contains

residual strain and displays severe twinning. In fact, the areal ratio between the two twins is about unity, implying equiprobable nucleation of each twin type.

V_2O_3 exhibits an even more pronounced thermal expansion between room temperature and 750°C of about 1.0 %[22]. While virtually perfectly lattice matched to Cr_2O_3 at room temperature, the high temperature misfit of Cr_2O_3 on V_2O_3 is about -0.5 %. When cooling to room temperature, the largest part of this differential strain of the V_2O_3/Cr_2O_3 is again stored in the underlayer, as the Cr_2O_3 film has a substantially larger volume. The differential Al_2O_3/V_2O_3 strain, however, affects both layers. The V_2O_3 lattice thus tries to contract about 0.5 % more than the Al_2O_3 lattice. As shown by the XRD data [**Supplementary Figure 3**(c)], only a small part of this thermal strain can still relax during the cool-down and the major part – about 0.3 % - is retained in the Cr_2O_3 lattice as an elastic c-axis compression. The strain stored in the V_2O_3 layer is expectedly even larger and amounts to 0.45 %.

Due to the subtle high temperature misfit, the V_2O_3 underlayer causes the lowest density of misfit dislocations in Cr_2O_3 with an average spacing of about 90 nm per misfit line. Compared to Cr_2O_3 on Pt, the linear misfit density is thus expected to be lower by a factor of about 6 using the V_2O_3 underlayer. The areal misfit density would be reduced by even the square of that factor. In further contrast to the Pt or Al_2O_3 underlayers, the misfit dislocations in the V_2O_3 system are expected to have an inverse stacking order, as the differential strain in the Cr_2O_3 layer is tensile at high temperatures with respect to the V_2O_3 lattice. At the same time, twinning is greatly suppressed when using a V_2O_3 underlayer as a gate electrode instead of a Pt layer [**Supplementary Figure 3**(f)]. The reason for this is that the corundum lattice is allowed to continuously extend from the substrate up to the Cr_2O_3 layer and no structure type boundary exists along the growth direction. Twinning mechanisms will be discussed in more detail below.

While the top surface of the magnetoelectric antiferromagnet is formed as a vacuum boundary during the annealing process, the situation for the bottom surface is more complex. The high temperature during annealing causes atom diffusion not only within Cr_2O_3, but potentially also an exchange of atoms between the underlayer and Cr_2O_3. Such interdiffusion occurs when Cr_2O_3 and the underlayer can form a solid solution or an alloy. Furthermore, during annealing at 750°C the Cr_2O_3 crystal relaxes any potential residual elastic strain apart from a small interfacial region, in which the interface bonds with the underlayer lattice disfavor the relaxed Cr_2O_3 lattice. As most of the Cr_2O_3 crystal tries to relax its stress, the misfit dislocations are transported to a region very close to its bottom interface, in case they formed further within the bulk.

When the underlayer is Pt, one potential effect is the dissolution of Cr into Pt. However, this process is strongly unfavorable in the case of Cr_2O_3 as, for Cr to dissolve, the oxide would need to be broken up and oxygen liberated. Therefore, a stoichiometrically sharp interface is expected between Cr_2O_3 and Pt[23]. In contrast, the Al_2O_3 or V_2O_3 underlayers are isostructural with Cr_2O_3 and are both oxides, which implies that Cr_2O_3 does not have to break up for intermixing to occur. Instead, the compounds mix by merely exchanging their trivalent metal cations. The miscibility depends on how similar the lattice constants are[24], implying more favorable and faster intermixing at V_2O_3/Cr_2O_3 interfaces. As the interfaces between the corundum oxides prepared at elevated temperatures can be considered a continued crystal with cation content gradually changing over some atomic distances[25,26], no atomically sharp interface exists. Therefore, the ferromagnetic boundary layer expected for free surfaces of Cr_2O_3 cannot arise in the same way at such interfaces.

While the particular effects differ between the various underlayers investigated here, the two unlike boundaries of the Cr_2O_3 layers introduce an inherent imbalance between the top and bottom ferromagnetic boundaries. As such magnetic surface effects are increasingly important when the film thickness is reduced, they must be considered when investigating the magnetic and magnetoelectric behavior of the thin Cr_2O_3 films. Eventually, ferrimagnetism as investigated throughout this manuscript can result from this magnetic moment imbalance.

The macroscopic degree of crystallographic twinning probed by XRD showed that the oxide thin films in all-corundum systems have a tendency to retain the 3-dimensional structural order of the Al_2O_3 substrate [**Supplementary Figure 3**]. The marked preference for one twin domain type over the other implies that the minority twins are stabilized merely by the presence of local defects that disturb the ideal Al_2O_3 surface. **Supplementary Figure 4** shows orientation contrast images to assess the microscopic pattern of the twin crystals. The contrast is obtained in an electron microscope by illuminating the surface along the oblique $[1\,0\,4]$ direction. Under such a high symmetry direction, the atomic lattice contains channels that allow the probe electrons to enter far into the material before creating secondary electrons[27]. For the twin grain, these channels are rotated by 60° in the film plane and are thus not directly irradiated by the probe electrons. As a result, the twin type with more pronounced channeling will release less secondary electrons as they are created deeper inside the material.

Supplementary Figure 4(a) was obtained for an $Al_2O_3/V_2O_3/Cr_2O_3$ sample with only little twinning. The brighter minority twins are randomly distributed and their areal density is homogeneous at scales large than a few 100 nm. However, they vary substantially in size from

below 10 nm up to 50 nm. As annealing the epitaxial film would rather remove small minority twin domains instead of creating them, the origin of the twinning must lie in the nucleation stage of film growth.

Cr_2O_3 films on non-corundum materials, on the contrary, reveal equiprobable twin domains as the seed layer exerts no preference for one particular epitaxial twin[8]. The twinning pattern then reveals the size of the individual islands that formed during the film growth. Due to the high mobility of Cr and O adatoms on (111) noble metal surfaces[23] the individual nucleations are substantially larger, than for Cr_2O_3 on other oxides, where bonding and sticking is stronger. As can be seen in **Supplementary Figure 4**(b) some Cr_2O_3 twin domains are over 100 nm in diameter. The image was taken for an Al_2O_3/Ni/Ag/Cr_2O_3 sample, which – like the Pt buffered films – contains twin domains of both types equiprobably.

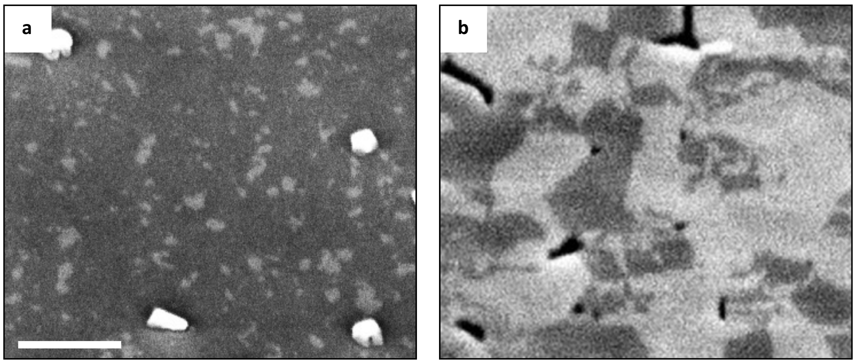

Supplementary Figure 4 | Electron channeling contrast micrographs recorded using an electron illumination along the $[1\ 0\ 4]$ direction of Cr_2O_3, which is tilted 31° off normal. The scale bar is 200 nm and the viewed area is a $800 \times 800\,\mathrm{nm}^2$ square **a,** Cr_2O_3 on a V_2O_3 underlayer shows an obvious preponderance of crystallographic twin domains with a dark contrast. **b**, Cr_2O_3 on a noble metal underlayer, in contrast, has a more equal occurrence of the two twin domain types.

Channeling contrast microscopy reveals that twinning is present in epitaxial Cr_2O_3(0001) films at scales of about 100 nm and below. On average, the observed twinning fraction found at such microscopic areas is consistent with the overall twin domain presence. Twinning does not affect the smoothness and continuity of epitaxial annealed Cr_2O_3 films. Therefore, twinned films are on equal footing with single orientation films from a structural point of view for the intents of this work.

As twinning evidently takes place on the scale of individual crystal nucleations regardless of the underlayer material, the approximate twin sizes are on the order of 100 nm. As the electrically probed regions are substantially larger with a diameter of about 10 µm, the twinning fraction

within these regions is expected to be consistent with that measured by XRD for the entire sample.

7. Theoretical background on using zero-offset Hall to probe ferrimagnetism

The magnetic moment present in otherwise AF thin films of Cr_2O_3 can generate an order parameter selection pressure even when both the electric field and exchange bias are zero ($E = 0$, $J_{\mathrm{EB}} = 0$). In this scenario, the film can be considered ferrimagnetic and the magnetic moment $m = \rho_m A$ contained in a particular area A can invoke thermally activated selection of the magnetic order parameter. As the expected magnetic moment only arises as a small deviation from perfect AF order, the magnetic susceptibility of the order parameter is small and rather strong magnetic fields of several $100\,\mathrm{kA\,m^{-1}}$ are necessary for stable order parameter selection at room temperature. In the present thin film samples, a distribution of magnetic moments $\mathcal{M}(m)$ is expected due to both a natural spread of the size of the individual areas and a dependence of the areal magnetic moment density on the ordering temperature. The order parameter selection preference as a function of the applied magnetic cooling field is then described as a convolution:

$$\eta(H_{\mathrm{cool}}) = \int_0^\infty \tanh\left(\frac{\mu_0 H_{\mathrm{cool}} m}{k_{\mathrm{B}} T_{\mathrm{crit}}}\right) \mathcal{M}(m)\,\mathrm{d}m \qquad (3)$$

The variation of the critical temperature among the individual domains can be neglected for the $k_{\mathrm{B}}T_{\mathrm{crit}}$ term as it has a spread of only about 1 % from its absolute mean value $\langle T_{\mathrm{crit}}\rangle = 299\,\mathrm{K}$. The influence of this spread on the areal magnetic moment density is expected to be larger, but is already taken care of by the distribution $\mathcal{M}(m)$. Therefore, T_{crit} will be regarded as a fixed parameter for this analysis. The distribution of the magnetic moments will be modeled by a Chi distribution

$$\mathcal{M}(m, \langle m\rangle, k) = \mathrm{X}\left(\frac{m}{\langle m\rangle}, k\right) \qquad (4)$$

which describes a positive variable generated by an unknown number of degrees of freedom k. The Chi distribution is selected here, because it is a very general distribution with only two parameters and has several relevant special cases such as the Normal, Rayleigh or Maxwell distributions.

Using Eq. (3), it is possible to construct fits to experimental data of the field-invariant zero-offset Hall signal in dependence of the magnetic cooling field $R_{\mathrm{inv}}(H_{\mathrm{cool}})$. This has been done for the

three main categories of samples [**Table 2**] and for one system without a Hall cross on top of the Cr_2O_3 layer. The latter was investigated by using the unpatterned Pt(20 nm) underlayer as a proximity magnet, which is possible due to the robust rejection of geometric Hall cross asymmetries of the zero-offset Hall technique[19]. The fits depend on three parameters, namely the two parameters $\langle m \rangle$ and k describing the Chi distribution of the magnetic moments and the saturation Hall resistance R_{N}.
The cooling field dependences [left column in **Supplementary Figure 5**] of all the studied systems are well accounted for by the developed model [Eq. (3)], which yields a representation of the data that is essentially free of systematic errors. While the top-measured Al_2O_3/Cr_2O_3/Pt and Pt/Cr_2O_3/Pt systems reveal a marked positive dependence – and thus a positive magnetic moment – the bottom-measured Pt/Cr_2O_3/air and the top-measured V_2O_3/Cr_2O_3/Pt systems show inverse cooling field dependences of the zero-offset Hall signal. This inversion is caused by a net negative moment with respect to the sign of the magnetization in the probed boundary layer. In this respect, it is noteworthy that the dependences measured at the top and bottom boundaries in the Pt/Cr_2O_3/Pt system, respectively, are highly similar [**Supplementary Figure 5**(d,f)] apart from the inversion. Indeed, the two systems are expected to be structurally very similar, as the only difference is the deposition of the top Pt layer at room temperatures after the annealing of the magnetoelectric antiferromagnet layer. Both boundaries of the Cr_2O_3 layers in the top-measured Pt/Cr_2O_3/Pt and the bottom-measured Pt/Cr_2O_3/air system are thus prepared under identical conditions. The sign inversion of the field-invariant zero-offset Hall signal is thus a clear fingerprint of the fact that different sublattices of the antiferromagnet dominate the top and the bottom boundary of the magnetoelectric antiferromagnet layer, which is indeed in line with theoretical predictions[28]. The striking similarity in the magnetic field behavior of the order parameter selection [**Supplementary Figure 5**(d,f)] also implies that the room temperature deposition of the thin top Pt layer after the annealing process of the Cr_2O_3 layers has a negligible influence on the magnetic properties of the top boundary. Furthermore, this result also indicates that the magnetic moment of the bottom boundary sublattice is larger because the bottom layer is observed to align along the magnetic cooling field, while the top layer is aligned antiparallel to the cooling field direction. This conclusion is based on the observedly negative anomalous Hall signal of the Cr_2O_3/Pt proximity system [**Supplementary Figure 1**].

Before discussing the influence of the three different underlayers and hence the three sample categories [**Table 2**], it is important to consider the meaning of the individual fitting parameters listed in the right column panels of **Supplementary Figure 5**. The saturation value of the field-

invariant zero-offset Hall signal R_N is affected by the magnetic moment in the magnetic boundary and by the quality and thickness of the proximity magnet. In the present systems, the magnetic moment of the magnetoelectric AF boundary layer can be assumed to be similar for the bottom and top termination, unbalanced only to a few percent. As all the magnetic layers measured by Hall are epitaxial Cr_2O_3/Pt interfaces, the structural configuration can be also assumed to be similar. The remaining influence on R_N is thus mainly related to the thickness induced signal shunting behavior of the Pt layer. This explains the about one order of magnitude lower value of R_N for the Pt/Cr_2O_3/air system in comparison to the other systems, because the Pt underlayer has a thickness of about 20 nm compared to about 2.5 nm for the Pt top layers used in the other systems. In contrast to R_N, which is determined by the properties of the interface probed by Hall, the parameters $\langle m \rangle$ and k describe the entire magnetic moment of the Cr_2O_3 layer over a particular area that selects an AF order parameter. The sign of $\langle m \rangle$ indicates if the total magnetic moment has the same or antiparallel orientation as the proximity magnetization. While all three parameters (R_N, $\langle m \rangle$ and k) are usually well confined [**Supplementary Figure 5**(b,d,f)], the measured dependence for the V_2O_3/Cr_2O_3/Pt system [**Supplementary Figure 5**(g)], is essentially linear with a rather low amplitude. This renders R_N and $\langle m \rangle$ redundant parameters and makes k badly determined. To obtain a meaningful interpretation, it is thus necessary to include the electric field as another stimulus [main text] and thus fix the saturation value R_N.

The general appearance of magnetic field susceptibility of the AF order parameter in all the studied magnetoelectric antiferromagnet thin film systems unambiguously demonstrates that the Cr_2O_3 films are not perfectly AF but have two slightly unbalanced sublattices giving rise to ferrimagnetism, even with no applied electric field. As there is no intrinsic cause for the broken sublattice equivalence, the parasitic magnetic moment must arise as a result of the extrinsic sample properties. Through careful review of the engineered differences between the three sample categories, it is possible to draw conclusions on which extrinsic properties drive the appearance the ferrimagnetism in magnetoelectric antiferromagnet thin films.

By measuring the zero field point in the dependences in **Supplementary Figure 5** many times, it is possible to determine the scatter induced due to the finite number of selectable domains within the Hall cross. This is shown in **Supplementary Figure 6**. The shown kernel density distributions are already deconvolved to remove the influence of the measurement accuracy. The remaining spread is purely a consequence of the domain discretization within the Hall cross and can be used directly to judge the relative domain sizes within the Cr_2O_3 layers on the

various underlayers. Therefore, it is possible to measure both the domain moment, and the domain sizes using zero-offset Hall, which allows one to derive the ferrimagnetic areal moment density in the films. For this manuscript, only relative domain sizes will be used as the conclusion does not depend on the absolute sizes.

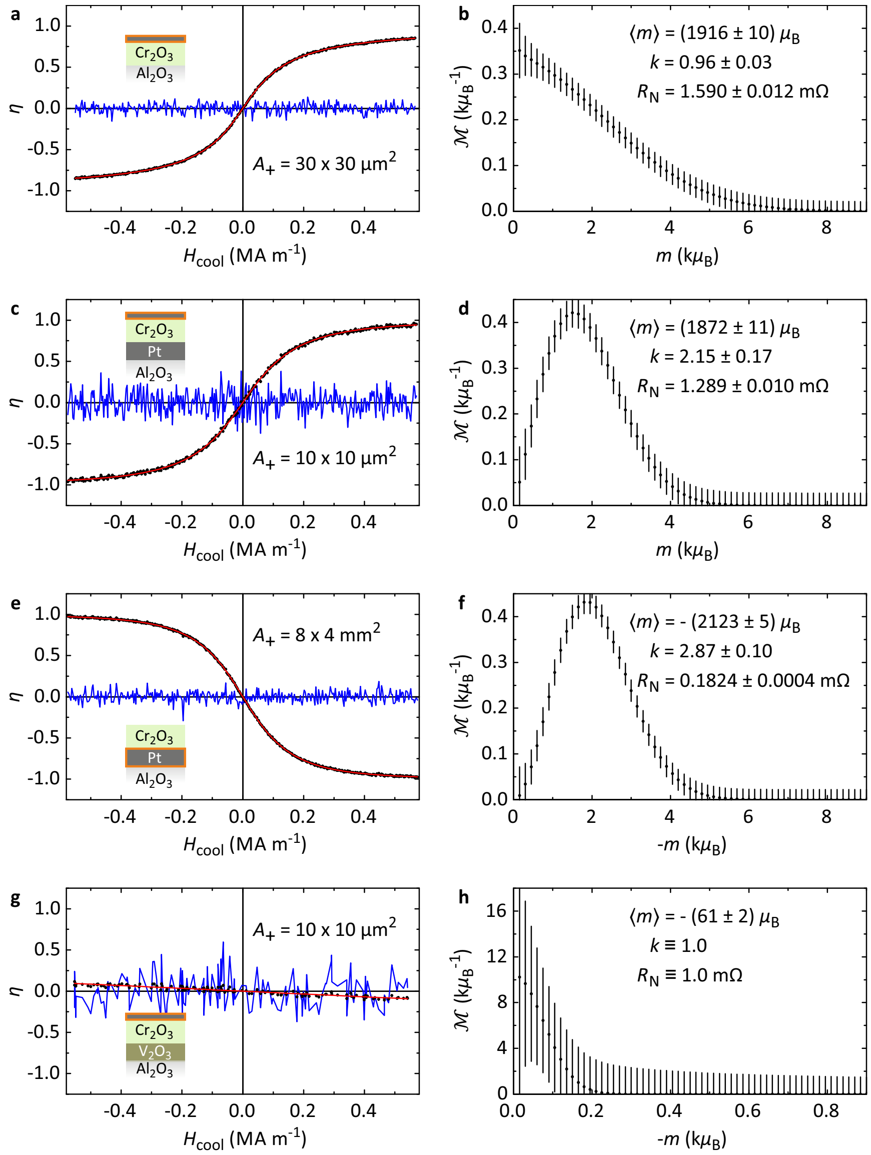

Supplementary Figure 5 | a,**c**,**e**,**g**, Experimental dependences of the AF order parameter on the magnetic cooling field for four different samples with different bounding layers as shown in the insets. Zero-offset Hall measurements have been carried out on the orange highlighted layer. Red lines show fits obtained using Eq. (3) and blue lines are residuals x10. **b**,**d**,**f**,**h**, Corresponding magnetic moment distributions as obtained from the fits.

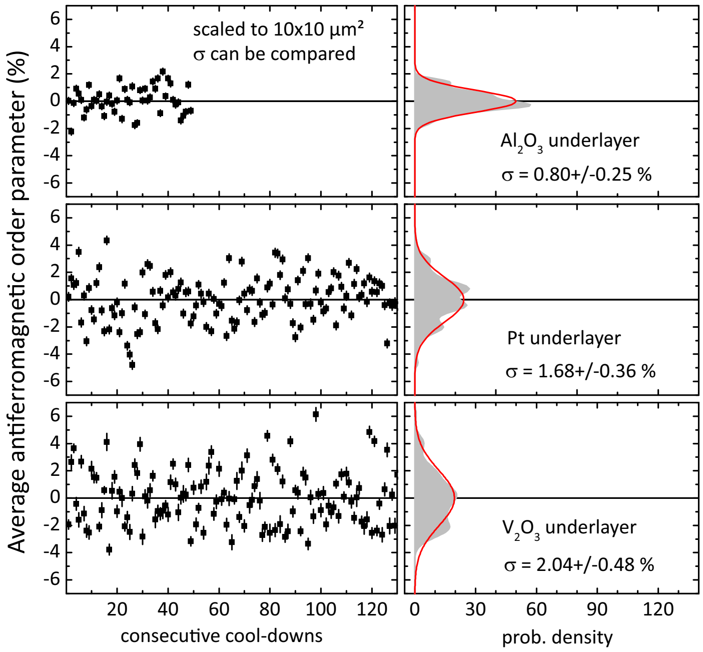

Supplementary Figure 6 | Relative domain size measurement. By studying the variations between identical cool-downs, one can conclude on the relative average domain sizes.

8. Positron Annihilation Spectroscopy

The fate of positrons in solids is to thermalize, diffuse, and then annihilate with core and valence electrons of the material, which results in emission of two ~511 keV gamma photons. Due to the momentum of the electrons, the variation from that value is a result of Doppler broadening (DB) of the annihilation line. The Doppler broadening is characterized by the shape parameter S and the wing parameter W. More details about both parameters can be found elsewhere[29,30], but in general the profile of these parameters in terms of positron implantation depth is influenced by the stopping profile $P(z, \epsilon)$ and positron diffusion. The S parameter is more sensitive to the open volume defects concentration and their size, whereas the W parameter is a fingerprint of the annihilation site surrounding.

Here, we employ a DB positron annihilation spectroscopy (PAS) setup for defect concentration depth profiling of a 170 nm thick epitaxial Cr_2O_3 film on an Al_2O_3 substrate (thickness determined by cross-sectional TEM). The depth sensitivity is given by the variation of positron incident energies. The positron stopping profiles are approximated by a Makhovian distribution and the mean positron penetration depth is $z_{\mathrm{mean}} = A\,\rho^{-1}\epsilon^{n}$, where ϵ is the positron energy, ρ is the material density, and the parameters A and n are material-related constants. z_{mean} has been calculated for Cr_2O_3 and can be found in **Supplementary Figure 7**(a) as the top scale. The maximum positron implantation depth is about $2 \cdot z_{\mathrm{mean}}$. The $S(\epsilon)$ curve indicates an increased

open volume at an implantation energy of 7 keV, which corresponds to $z_{\mathrm{mean}} \approx 162\ \mathrm{nm}$, thus very close to the expected interface position. The black line at $\epsilon = 5\ \mathrm{keV}$ bounds the regime, in which basically all positrons annihilate in the Cr_2O_3 film only. The $S(\epsilon)$ dependence has been fitted with the VEPFIT code[31] in order to calculate the effective diffusion lengths in the system as well as the characteristic S-parameters. The fit shows a good agreement with experimental data.

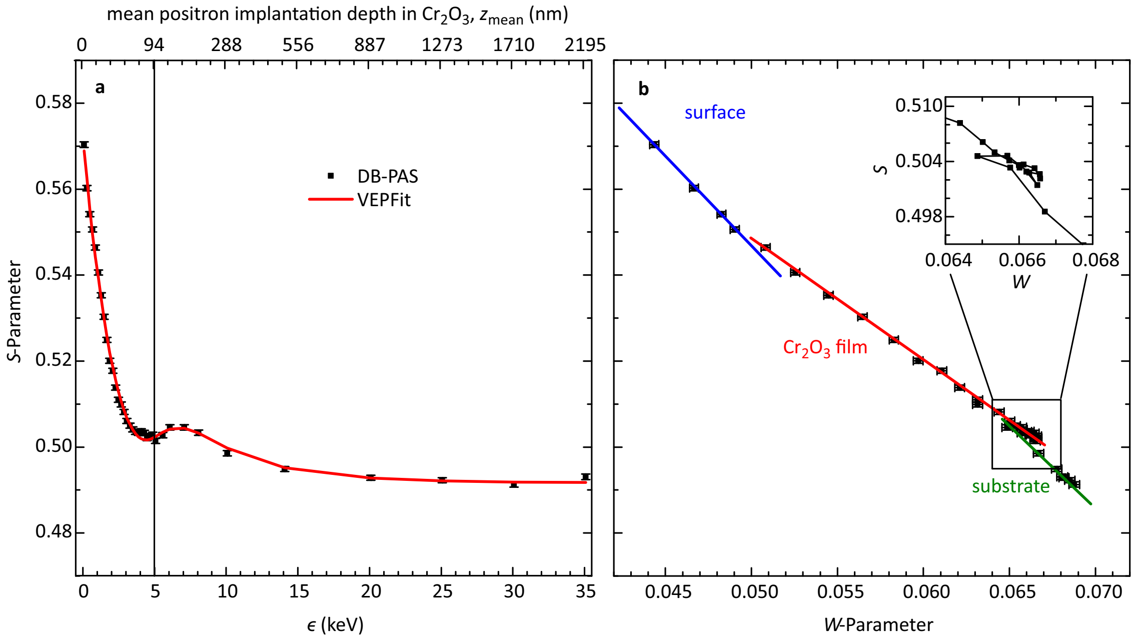

Supplementary Figure 7 | Positron Annihilation Spectroscopy of Cr_2O_3 thin films. a, Annihilation line parameter S, as a function of positron energy ϵ (black squares) with corresponding positron diffusion modeling (red line) extracted from the VEPFIT code. b, S-W plot clearly showing three distinct regions across sample thickness, namely a thin surface region, bulk, and a substrate. In the inset, the interface between the film and substrate is shown in more detail – the characteristic zig-zag shape of the curve indicates increased open volume at the interface.

Model 5 of VEPFIT is used to fit the data, where a rectangular distribution of defects and a layered structure are assumed. Four distinct regions have been considered, three of them with thicknesses of 10 nm, 159 nm, and 1 nm for a film-surface region, film-bulk, and film-interface, respectively. The fourth region is the substrate. The film-surface region has been recognized from the S-W plot [**Supplementary Figure 7**(b)], where the four data points corresponding to the lowest implantation energy lie on a slope different than that of the data taken at higher energies. This suggests a different defect type in the topmost 10 nm of the film, according to the Makhovian distribution. The other distinct regions are well discernible in the S-W plot, too. The

interface between the Cr_2O_3 film and the substrate is especially characteristic [inset of **Supplementary Figure 7**(b)], with a zig-zag shape of the S-W curve clearly visible. All the characteristic regions of the S-W plot can be fitted with lines of different slopes, which translates to different defect types. The fitting zone for the VEPFIT calculation is divided into 49 depth intervals starting with 0.1 nm and with an increment factor of 1.3. The best fit values for the S parameters are summarized in **Supplementary Table 1**. The epithermal scattering length is given at (2.5 ± 0.08) nm. The effective positron diffusion lengths for each of the four regimes have been fixed in the calculation yielding the best fit for values of about 5 nm, 20 nm, 1 nm, and 60 nm for the film-surface-region, film-bulk, film-interface, and the substrate, respectively. The diffusion length for the substrate is in agreement with previous reports[32]. The calculated S-parameters of the layers reveal a significantly enhanced trapping potential at the film-interface region, corresponding to an enhanced defect concentration at the Cr_2O_3/Al_2O_3 interface than in the bulk of the Cr_2O_3 film. The positron trapping in the film is likely due to mono-vacancies that are difficult to detect with other measurement techniques.

	surface	film-surface	film-bulk	film-interface	substrate
S-parameter	0.5452 (±.0020)	0.5405 (±0.0014)	0.49359 (±0.00031)	0.5378 (±0.0018)	0.49150 (±0.00029)
diffusion length (nm)		5	20	1	60

Supplementary Table 1 | S-parameters and diffusion lengths of distinct layers in the Cr_2O_3/Al_2O_3 system calculated by VEPFIT.

Supplementary References

1. Mu, S., Wysocki, A. L. & Belashchenko, K. D. Effect of substitutional doping on the Néel temperature of Cr_2O_3. *Phys. Rev. B* **87**, 054435 (2013).
2. Street, M. *et al.* Increasing the Néel temperature of magnetoelectric chromia for voltage-controlled spintronics. *Appl. Phys. Lett.* **104**, 222402 (2014).
3. Shiratsuchi, Y. *et al.* Detection and In Situ Switching of Unreversed Interfacial Antiferromagnetic Spins in a Perpendicular-Exchange-Biased System. *Phys. Rev. Lett.* **109**, 077202 (2012).
4. Lim, S.-H. *et al.* Exchange bias in thin-film $(Co/Pt)_3/Cr_2O_3$ multilayers. *J. Magn. Magn. Mater.* **321**, 1955–1958 (2009).
5. Shiratsuchi, Y., Fujita, T., Oikawa, H., Noutomi, H. & Nakatani, R. High Perpendicular Exchange Bias with a Unique Temperature Dependence in Pt/Co/α-Cr_2O_3(0001) Thin Films. *Appl. Phys. Exp.* **3**, 113001 (2010).
6. Shiratsuchi, Y. *et al.* High-Temperature Regeneration of Perpendicular Exchange Bias in a Pt/Co/Pt/α-Cr_2O_3/Pt Thin Film System. *Appl. Phys. Exp.* **6**, 123004 (2013).
7. Ashida, T. *et al.* Isothermal electric switching of magnetization in Cr_2O_3/Co thin film system. *Appl. Phys. Lett.* **106**, 132407 (2015).
8. Shiratsuchi, Y., Nakatani, T., Kawahara, S. & Nakatani, R. Magnetic coupling at interface of ultrathin Co film and antiferromagnetic Cr_2O_3(0001) film. *J. Appl. Phys.* **106**, 033903 (2009).
9. Toyoki, K. *et al.* Magnetoelectric switching of perpendicular exchange bias in Pt/Co/α-Cr_2O_3/Pt stacked films. *Appl. Phys. Lett.* **106**, 162404 (2015).
10. Ashida, T. *et al.* Observation of magnetoelectric effect in Cr_2O_3/Pt/Co thin film system. *Appl. Phys. Lett.* **104**, 152409 (2014).
11. Nozaki, T. *et al.* Positive exchange bias observed in Pt-inserted Cr_2O_3/Co exchange coupled bilayers. *Appl. Phys. Lett.* **105**, 212406 (2014).
12. He, X. *et al.* Robust isothermal electric control of exchange bias at room temperature. *Nat. Mater.* **9**, 579–585 (2010).
13. Toyoki, K. *et al.* Switching of perpendicular exchange bias in Pt/Co/Pt/α-Cr_2O_3/Pt layered structure using magneto-electric effect. *J. Appl. Phys.* **117**, 17D902 (2015).
14. Martin, T. & Anderson, J. Antiferromagnetic domain switching in Cr_2O_3. *IEEE Trans. Magn.* **2**, 446–449 (1966).
15. Wu, N. *et al.* Imaging and Control of Surface Magnetization Domains in a Magnetoelectric Antiferromagnet. *Phys. Rev. Lett.* **106**, 087202 (2011).
16. Folen, V. J., Rado, G. T. & Stalder, E. W. Anisotropy of the Magnetoelectric Effect in Cr_2O_3. *Phys. Rev. Lett.* **6**, 607–608 (1961).
17. Fiebig, M. Revival of the magnetoelectric effect. *J. Phys. D: Appl. Phys.* **38**, R123 (2005).
18. Fallarino, L., Berger, A. & Binek, C. Magnetic field induced switching of the antiferromagnetic order parameter in thin films of magnetoelectric chromia. *Phys. Rev. B* **91**, 054414 (2015).
19. Kosub, T., Kopte, M., Radu, F., Schmidt, O. G. & Makarov, D. All-electric access to the magnetic-field-invariant magnetization of antiferromagnets. *Phys. Rev. Lett.* **115**, 097201 (2015).
20. *Single Crystal Sapphire*. (Kyocera Corp. Fine Ceramics Group: 2014).at <http://global.kyocera.com/prdct/fc/product/pdf/s_c_sapphire.pdf>
21. Edsinger, R. E., Reilly, M. L. & Schooley, J. F. Thermal Expansion of Platinum and Platinum–Rhodium Alloys. *J. Res. Natl. Bur. Stand.* **91**, 333–356 (1986).
22. Eckert, L. J. & Bradt, R. C. Thermal expansion of corundum structure Ti_2O_3 and V_2O_3. *J. Appl. Phys.* **44**, 3470–3472 (1973).

23. Zhang, L., Kuhn, M. & Diebold, U. Epitaxial growth of ultrathin films of chromium and its oxides on Pt (111). *J. Vac. Sci. Tech. A* **15**, 1576–1580 (1997).
24. Kim, S. S. & Sanders, T. H. Thermodynamic Modeling of the Isomorphous Phase Diagrams in the Al_2O_3–Cr_2O_3 and V_2O_3–Cr_2O_3 Systems. *J. Am. Ceram. Soc.* **84**, 1881–1884 (2001).
25. Bayati, M. *et al.* Domain epitaxy in TiO_2/α-Al_2O_3 thin film heterostructures with Ti_2O_3 transient layer. *Appl. Phys. Lett.* **100**, 251606 (2012).
26. Kaneko, K., Kakeya, I., Komori, S. & Fujita, S. Band gap and function engineering for novel functional alloy semiconductors: Bloomed as magnetic properties at room temperature with α-$(GaFe)_2O_3$. *J. Appl. Phys.* **113**, 233901 (2013).
27. Dehm, G., Inkson, B. & Wagner, T. Growth and microstructural stability of epitaxial Al films on (0001) α-Al_2O_3 substrates. *Acta Mater.* **50**, 5021–5032 (2002).
28. Belashchenko, K. D. Equilibrium Magnetization at the Boundary of a Magnetoelectric Antiferromagnet. *Phys. Rev. Lett.* **105**, 147204 (2010).
29. Liedke, M. O. *et al.* Open volume defects and magnetic phase transition in Fe60Al40 transition metal aluminide. *J. Appl. Phys.* **117**, 163908 (2015).
30. Saleh, A. S. Analysis of positron profiling data by ROYPROF, VEPFIT, and POSTRAP4 codes: a comparative study. *J. Theo. Appl. Phys.* **7**, 1–6 (2013).
31. Van Veen, A., Schut, H., De Vries, J., Hakvoort, R. & Ijpma, M. Analysis of positron profiling data by means of 'VEPFIT. *4th International workshop on: Slow-positron beam techniques for solids and surfaces* **218**, 171–198 (1991).
32. Gordo, P. M. *et al.* On the defect pattern evolution in sapphire irradiated by swift ions in a broad fluence range. *Applied Surface Science* **255**, 254–256 (2008).
